# Open-sourced Raman spectroscopy data processing package implementing a baseline removal algorithm validated from multiple datasets acquired in human tissue and biofluids

**DOI:** 10.1117/1.JBO.28.2.025002

**Published:** 2023-02-21

**Authors:** Guillaume Sheehy, Fabien Picot, Frédérick Dallaire, Katherine Ember, Tien Nguyen, Kevin Petrecca, Dominique Trudel, Frédéric Leblond

**Affiliations:** a Polytechnique Montréal, Department of Engineering Physics, Montreal, Quebec, Canada; b Centre de recherche du Centre hospitalier de l’Université de Montréal, Montreal, Quebec, Canada; c McGill University, Montreal Neurological Institute-Hospital, Division of Neuropathology, Department of Pathology, Montreal, Quebec, Canada; d Institut du cancer de Montréal, Montreal, Quebec, Canada; e Université de Montréal, Department of Pathology and Cellular Biology, Montreal, Quebec, Canada; f Center Hospitalier de l’Université de Montréal, Department of Pathology, Montreal, Quebec, Canada

**Keywords:** Raman spectroscopy, fluorescence, tissue optics, open-sourced software, machine learning, optics

## Abstract

**Significance:**

Standardized data processing approaches are required in the field of bio-Raman spectroscopy to ensure information associated with spectral data acquired by different research groups, and with different systems, can be compared on an equal footing.

**Aim:**

An open-sourced data processing software package was developed, implementing algorithms associated with all steps required to isolate the inelastic scattering component from signals acquired using Raman spectroscopy devices. The package includes a novel morphological baseline removal technique (BubbleFill) that provides increased adaptability to complex baseline shapes compared to current gold standard techniques. Also incorporated in the package is a versatile tool simulating spectroscopic data with varying levels of Raman signal-to-background ratios, baselines with different morphologies, and varying levels of stochastic noise.

**Results:**

Application of the BubbleFill technique to simulated data demonstrated superior baseline removal performance compared to standard algorithms, including iModPoly and MorphBR. The data processing workflow of the open-sourced package was validated in four independent in-human datasets, demonstrating it leads to inter-systems data compatibility.

**Conclusions:**

A new open-sourced spectroscopic data pre-processing package was validated on simulated and real-world in-human data and is now available to researchers and clinicians for the development of new clinical applications using Raman spectroscopy.

## 1 Introduction

Over the last decade, Raman spectroscopy has seen a resurgence in biomedical applications, in good part due to its synergy with emerging advances in data interpretation enabled by recent trends in machine learning and artificial intelligence.^1^^–^^3^ The increasing appeal for Raman spectroscopy in medical applications can be traced back to the fact it allows non-destructive (e.g., non-ionizing radiation) interrogation of any biological tissue or fluid, potentially informing on hundreds of biomolecular vibrational bonds within the same measurement. A strength of the technique is that this information can be reinterpreted as a molecular fingerprint lending an interpretation of the material’s composition in terms of the relative concentration of proteins and specific amino acids, lipids, deoxyribonucleic acid, and ribonucleic acid, as well as water and other metabolites.^4^^,^^5^

In biomedical sciences, Raman spectroscopy technologies have been deployed at different spatial scales on tissues and on biofluids. Spatial scales consist of confocal microscopy applications for imaging at cellular resolution,^6^^–^^8^ up to mesoscopic scales for biofluids,^9^^–^^13^ *in situ* tissue measurements during surgery,^14^^–^^16^ and more recently, at macroscopic scales.^17^^–^^19^ For these applications, tissue and fluids were submitted to different pre-processing methodologies. These include *in situ in vivo* (without sample pre-processing), *in situ ex vivo* (aqueous solution to maintain tissue viability/integrity), deposition on *ex vivo* microscope slides for formalin-fixed, paraffin-embedded tissue, and centrifugation for blood and saliva applications. Other important aspects to consider, in all study designs and imaging protocols, relating to the potential impacts associated with measurement temperature,^20^ heat-induced damages, and signal contamination from preservation chemicals and contaminants.

Raman spectroscopy technology variants include spatial offset Raman spectroscopy (SORS),^21^^–^^26^ surface-enhanced Raman spectroscopy (SERS),^27^^–^^31^ and shifted-excitation Raman difference spectroscopy (SERDS).^32^^–^^34^ By modulating the distance between the excitation source and the light re-emission detection locations, SORS allows access to different tissue depths, albeit at the cost of reduced photonic signals, capitalizing on the varying photon sensitivity functions (co-called diffusion banana shapes) in highly scattering media. SERS uses the plasmonic effect from the coupling of Raman-active molecular bonds with nanostructured metallic surfaces to enhance the inelastic scattering signal of specific molecular bonds, effectively amplifying signals up to several orders of magnitude, although enhancement factors attained in biological material are usually more modest. Finally, SERDS is a method alleviating the need to remove baseline spectral contributions through a direct subtraction of spectra acquired at two closely separated excitation wavelengths. While this technique offers an attractive alternative to the use of background removal algorithms, it does not help resolve the issue of the limited signal-to-background ratio (SBR) (i.e., inelastic scattering over intrinsic tissue fluorescence) in bio-Raman spectroscopy. Fluorescence from biomolecules usually diminish monotonically (approximately quadratically) with excitation wavelength (λ), while the inelastic scattering cross section decreases approximately as $\lambda^{-4}$. Excitation wavelengths used in Raman spectroscopy are most often in the red to near-infrared (NIR) range, frequently including 670, 785, 830, and 1064 nm.^35^ The latter could, by probing tissue into the short-wavelength infrared (SWIR) region, provide advantages in terms of deeper tissue sensing (reduced elastic scattering and absorption) as well as an improved Raman-to-fluorescence ratio. However, these advantages need to be balanced against the need to switch sensing technologies, from CCD detectors to InGaAs detection technologies in the SWIR, at the cost of reduced sensor quantum efficiencies and usually less favorable noise characteristics. Time-resolved detection using pulsed lasers and time-gated measurements can also be used to address the signal-to-background problem in biological samples. This is done by limiting light detection to non-resonant interaction phenomena with relaxation time scales occurring on sub-nanosecond timescales, thereby excluding contributions from resonant phenomena such as fluorescence.^36^

**Fig. 1:**
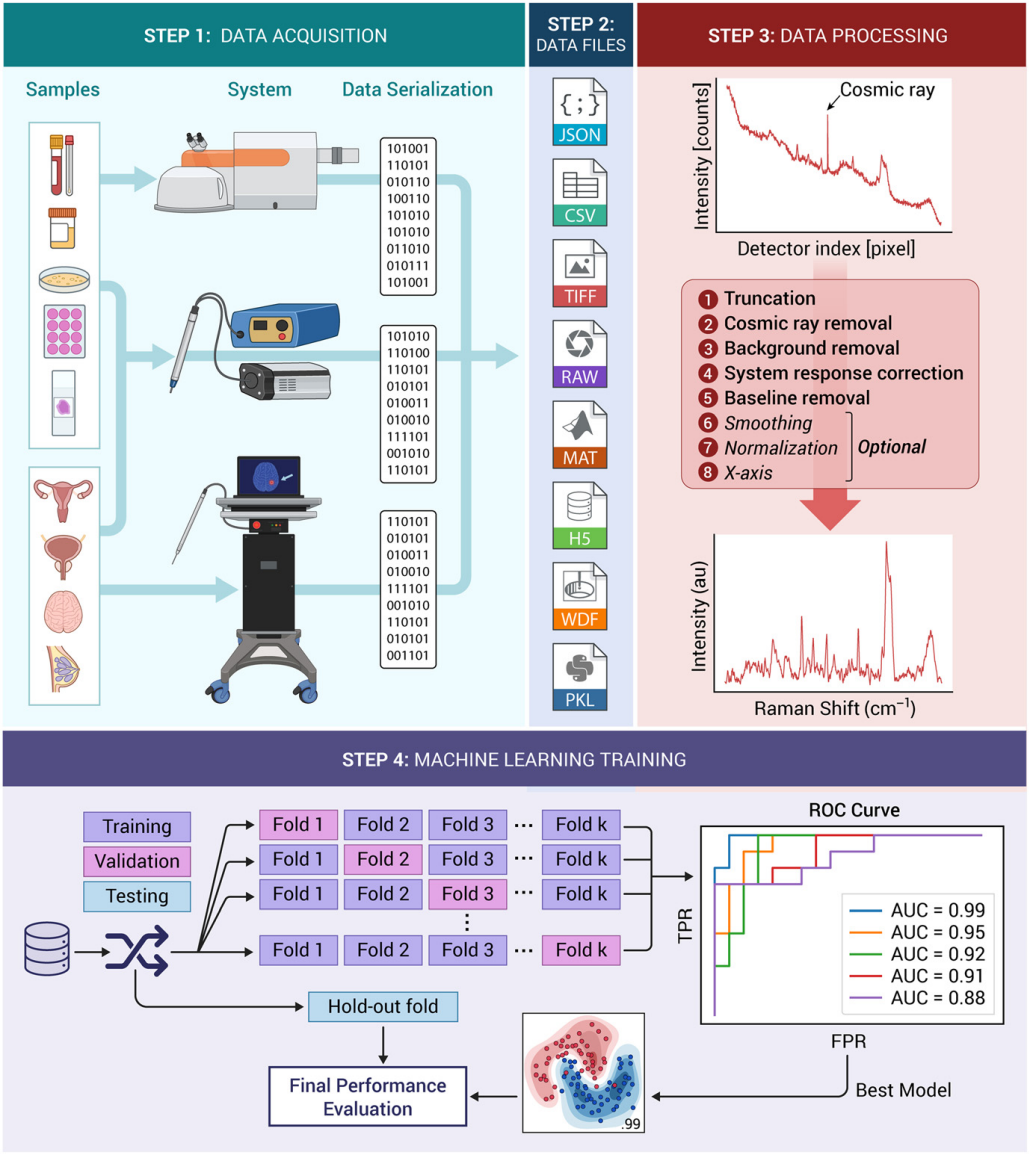
Depiction of all steps involved in acquiring spectroscopic data using Raman spectroscopy devices and the development of predictive machine learning models. STEP 1: spectra are acquired in different contexts [different organs (*in situ*, *ex vivo*, or fixed) or body fluids] with different instruments, including commercial Raman microscopes, hand-held surgical guidance probes or optical biopsy needles. STEP 2: Data (Raman spectra and metadata, including patient information) is stored in files with formats that can be processed using readily available tools (e.g., Python, Matlab). STEP 3 illustrates all pre-processing algorithms include in the open-sourced data processing package. STEP 4 illustrates the process involved in training, validation and testing of machine learning models with performance assessment based on receiver-operating-characteristic (ROC) analysis.

The common denominator of most of these Raman spectroscopy methods is that they lend spectra composed of up to 1000 to 2000 intensity bins (e.g., vectors) that require post-acquisition processing data treatment to isolate the inelastic scattering contribution associated with the interrogated material. This is essential because the inelastic scattering (i.e., Raman scattering) contribution is typically orders of magnitude smaller when compared to endogenous fluorescence from tissue biomolecules (e.g., collagen, elastin, NADH, and FAD). Moreover, spectra are always distorted by instrument signal contributions (e.g. fluorescence and Raman scattering from optical components), by the spectrally varying response of its optical components, as well as the presence of cosmic rays.^37^^–^^39^ Post-acquisition processing is generally performed as an intermediary step, after spectral acquisition and before (hence pre-processing) visual analysis or machine learning applications (Fig. 1). In that context, achieving Raman signal pre-processing in a manner that is standardized and that requires minimal expert knowledge is essential for the future development of Raman spectroscopy biomedical applications. This would allow researcher to compare their processed data (i.e., Raman spectra), would enable compatibility and portability of machine learning models developed for similar biomedical applications, and ensure future proofing of currently developed technologies.

This manuscript introduces the open Raman processing library (ORPL), an open-sourced spectral processing python package that includes a new morphological baseline (e.g., fluorescence from bio-molecules) removal algorithm named BubbleFill. This novel algorithm features several advantages over other state-of-the-art methods, such as a reduced reliance on expert knowledge and a decreased risk of under- and over-fitting spectral curves. The library comes with a module allowing to generate benchmark spectra with various Raman, baseline and noise characteristics. The spectra generated from this tool, along with real-world datasets, acquired on different tissue/fluid types using different instruments, are used to demonstrate the use of techniques and the low-level of variability induced by changes in level and shape of the fluorescence baseline and instrument response.

## 2 Methods

The ORPL, (pronounced “orpel”) package offers the necessary tools for processing Raman signals acquired with a variety of different system types, and was optimized to address the specific challenges that arise with biological samples. As there are currently no standards defining which methods should be used for pre-processing steps (both in academia and industry), systems developers tend to include proprietary and obfuscated algorithms and spectral processing techniques within systems controllers and data acquisition software. In some cases, it is neither possible nor practical (without extensive software modifications) to export and save a raw spectrum measured by the detector (prior to e.g., cosmic ray removal, averaging, filtering, and instrument response correction). This renders unrealizable the use of a unified processing workflow across Raman datasets measured with different instruments. For this reason, ORPL was designed to be modular, with each module being independent of the others, allowing out-of-sequence usage.

**Fig. 2:**
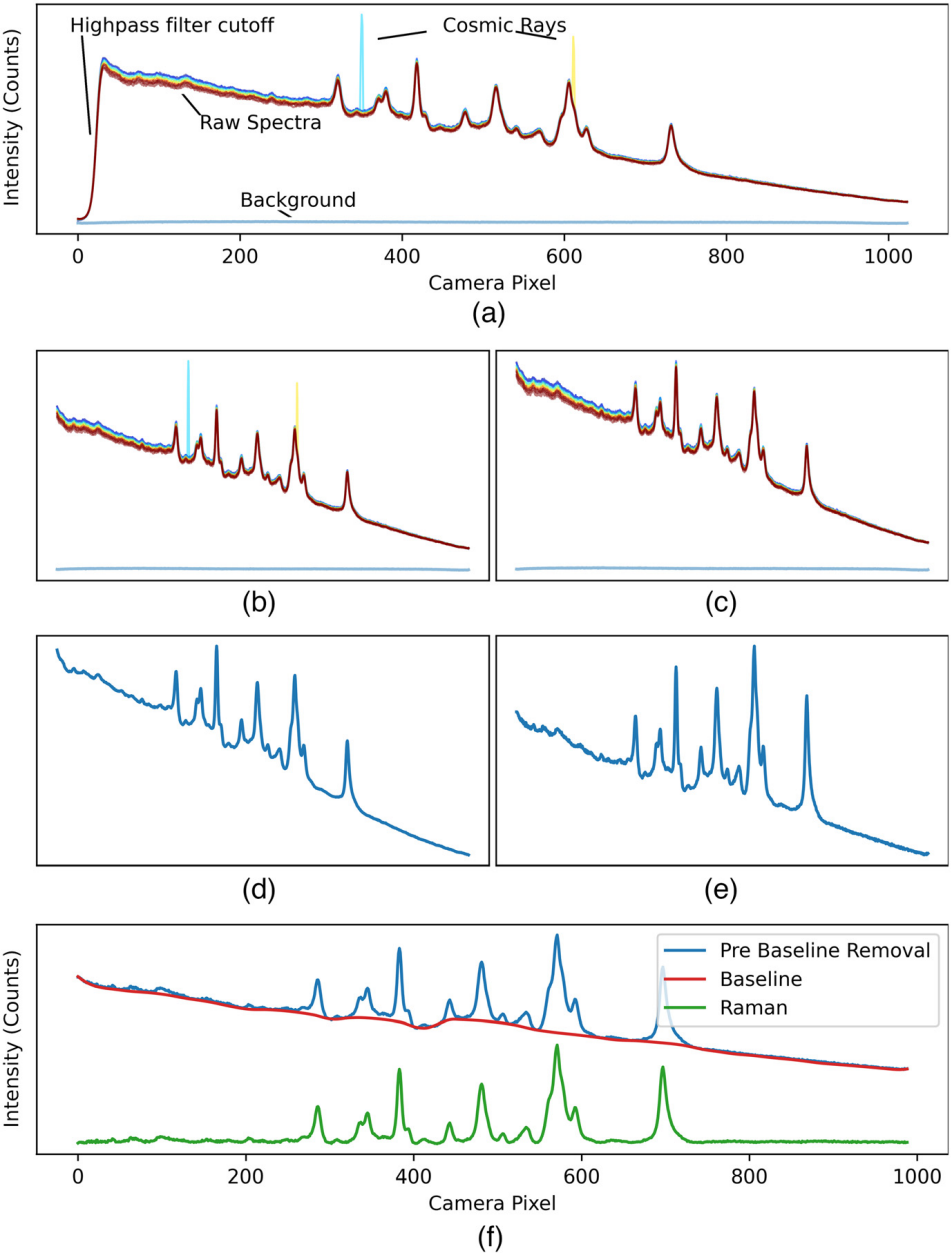
Overview of processing steps demonstrated on a signal measured from nylon with a point-probe system. (a) The raw accumulations and background; (b) after truncation; (c) after cosmic ray removal; (d) after background removal and combining accumulations; (e) after y-axis calibration; and (f) after baseline removal.

This section presents the different modules and tools that are available as part of the ORPL package, and the general guidelines for Raman signal pre-processing that our group has developed through multiple studies since 2013 [Fig. 1(a)]. An example of spectral processing is shown in Fig. 2, where each intermediary step is illustrated. The acquisition was performed on a sample of Nylon with a spectrometer from the company EMVision (equipped with a Newton CCD camera, Andor) coupled to a 785-nm laser (Innovative Photonics Solutions). Spectra were measured using a handheld probe (EMVision) and the system was operated via a custom in-house software. First, a single spectrum was measured with the excitation source turned off, that is the background signal (e.g., including ambient light). Then, a series of N spectra was measured with the excitation source turned on; those are the accumulations or raw spectra. Additional measurements were made on a acetaminophen tablet and on a NIST SRM 2241 standard for x-axis and y-axis calibration, respectively. The processing steps are applied in the following order: truncation (Sec. 2.1), cosmic ray removal (Sec. 2.2), background removal and combination of the accumulations (Sec. 2.3), y-axis calibration (Sec. 2.4) and baseline removal (Sec. 2.5). Additional steps may include x-axis calibration (Sec. 2.6), smoothing and normalization (Sec. 2.7), but are optional and depend on end-usage of the measured Raman spectrum. Quantitative metrics can also be used to quantify the spectral quality of the spectra to allow comparison between spectra acquired with different systems in the scope of different studies (Sec. 2.8).

### 2.1 Truncation

Raman spectrometers are frequently outfitted with high-pass filters (e.g., interference optical filters) to remove Rayleigh-scattered light that is typically orders of magnitude more intense than inelastic scattering. The filter cutoff must be as sharp and as close as possible to the excitation wavelength to limit excitation light bleed-through while maintaining sensitivity to Raman shifts <400 to $500\;{\mathrm{cm}}^{-1}$. At the same time, the acquisition spectrometer window may extend past the filter cutoff point, toward shorter wavelengths. This results in a spectral shift region at the beginning of every measured signal where the high-pass filter transition and the spectrometer window overlap [Fig. 2(a)], camera pixels 0–50). This region is truncated and removed from the measured accumulations and background.

### 2.2 Cosmic Ray Removal

Cosmic rays randomly hit spectrometer camera pixels during the acquisition of Raman signals, resulting in the appearance of sharp artifacts. Short of reducing exposure time, nothing can be done to mitigate the presence of cosmic rays during acquisitions. Worse, the typically long acquisition times required for biological samples (seconds, up to minutes in some cases) makes the presence of cosmic ray artifacts likely. Fortunately, they are relatively easy to remove using one of the following methods.

The first approach, implemented in ORPL’s crfilter_single() function, relies on the localized nature of the cosmic ray artifact. Since their spectral span is usually limited to 3–5 camera pixels, it is possible to (1) use the numerical derivative of the spectrum, (2) identify cosmic ray artifacts using an adaptive threshold, and (3) remove the artifact from the original spectrum using interpolation. This method should be limited to cases where it is impossible to acquire several spectra during an acquisition (i.e., a single accumulation, N=1), or for removal of cosmic rays from a background signal. This is because it can be difficult to tune the algorithm parameters to remove every cosmic ray while keeping the signal of interest intact.

The second approach, implemented in ORPL’s crfilter_multi() function, relies on the random nature of cosmic rays. Because it is exceedingly unlikely that two spectra or accumulations exhibit a cosmic ray artifact at the same wavelength (same camera pixel), it is possible to (1) compare intensity across spectra (from different accumulations) wavelength per wavelength to compute the disparity, (2) identify outliers as artifacts, and (3) replace the region neighboring the artifact using interpolation. Related spectra can be the different accumulations from a single site using a point-probe system, or spectra acquired over a small sample region using an imaging system such as a Raman microscope.

In general, the second method should be prioritized whenever possible, as it tends to identify cosmic rays more reliably while minimally affecting the filtered signals and is easier to tune. However, it can only be used effectively on multiple signals or accumulations, which are not always available.

### 2.3 Background Removal and Combining Accumulations

Backgrounds are measured before an acquisition to account for contributions to the signals that are not related to the sample, such as ambient light. They should be measured with the same experimental parameters (e.g., exposure time and instrument position whenever possible) as the main acquisitions, but with the excitation source turned off. It is also important to make sure no cosmic ray artifacts are present on a background spectrum to avoid introducing a downward spike in the signal. This can be done using the crfilter_single() on the background before removal when a single background signal is measured, or with crfilter_multi() otherwise. After this, the background is directly subtracted from the spectrum, after normalizing for exposure time. When multiple accumulations are measured for the same acquisition site, they are combined into a unique spectrum by computing their arithmetic mean, leading to improved signal-to-noise ratio (SNR). If that is the case, the background should be subtracted after the accumulation average to avoid unnecessary computational steps.

### 2.4 Y-axis Calibration

Instrument response correction is a necessary step for any spectroscopic measurement. Typical methods involve measuring using a spectrum from a reference calibration light source of known emission profile to recover the instrument response function (IRF). However, this approach can be challenging to integrate in most Raman spectroscopy acquisition workflows due to the requirement of additional equipment and the difficulty of positioning the calibration lamp.^40^ Instead, an alternate method for calibrating Raman instruments (e.g., correct for filter, detector etaloning, and quantum efficiency effects^41^) is based on measurement on a standard reference material (SRM) calibrated and manufactured by NIST (SRM-2241 for 785-nm excitation).^35^ Because this method does not involve the use of additional instruments (irradiance source), it is better suited for use in clinical environments where time and space are limited. Additionally, reference materials can be encased in a custom 3D-printed enclosure to facilitate positioning of probes or instruments and improve systems calibration repeatability. With this method, the IRF is computed from a Raman spectrum measured on the SRM using experimental parameters (laser power and exposure time) that maximize the sensor’s dynamic range. Then, the measured spectrum is processed using the steps previously described: truncation, cosmic ray removal, background removal. After those steps, the instrument’s IRF is computed from the known theoretical fluorescence response (${\mathrm{SRM}}_{\text{theoretical}}$), given as polynomial coefficients by the manufacturer and the measured signal (${\mathrm{SRM}}_{\text{measured}}$) as

$$\mathrm{IRF}=\frac{{\mathrm{SRM}}_{\text{measured}}}{{\mathrm{SRM}}_{\text{theoretical}}}.$$ (1)

The instrument response is then corrected for by dividing the Raman spectrum acquired by the IRF vector.

### 2.5 Baseline Removal

#### 2.5.1 Standard algorithms

**Fig. 3:**
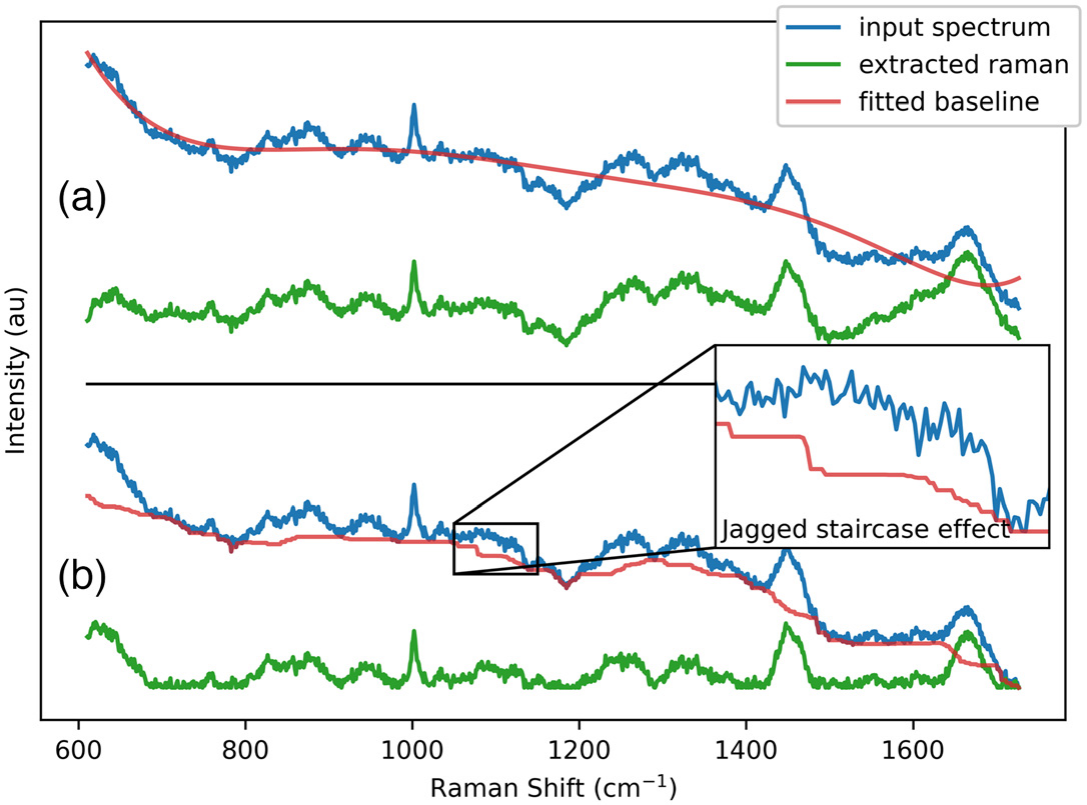
Demonstration of problems with common baseline removal algorithms. The spectrum used in this example was measured with a Renishaw Raman microscope on saliva samples.^13^ (a) iModPoly (polynomial fit) and (b) MorphBR (morphological baseline removal).

Baseline removal is the most difficult yet most critical step in the processing of a Raman spectrum. Because the probability of Raman scattering is orders of magnitude smaller compared to elastic scattering and fluorescence (in biological materials), even small artifacts introduced during this step can have a disastrous effect and completely overshadow the Raman signal. A common baseline removal method consists in using a polynomial fit (typically of order 5 or 6) coupled with a peak rejection rule (e.g., iModPoly algorithm^42^). The peak rejection rule is used to exclude regions of the signal that feature Raman peaks. Ideally, the entire Raman signal is excluded, leaving only a smooth baseline to be fitted with the polynomial function. This method is simple and can be effective in cases where Raman peaks are easily detected with simple peak-finding algorithms and if the baseline can be well-modeled by a polynomial function. However, it is not often the case for biological samples. Although intrinsic fluorescence can be adequately modeled as a polynomial function, the same cannot be said for absorption and scattering within the visible-IR range. Furthermore, biological Raman peaks are weak and complex, which makes them far harder to automatically detect compared to pure chemical compounds or inorganic materials. The result is that polynomial methods for baseline removal are difficult to tune and often introduce important artifacts when used for spectra measured on biological samples [Fig. 3(a)]. A method developed by Perez-Pueyo based on morphological processing (referred to as MorphBR moving forward) has shown to be better suited than polynomial fitting for biological applications.^43^ However, baselines removed using this algorithm feature a jagged staircase effect and a “hill,” that is as wide as the filtering window, near the beginning of the spectrum [Fig. 3(b)].

#### 2.5.2 BubbleFill algorithm

**Fig. 4:**
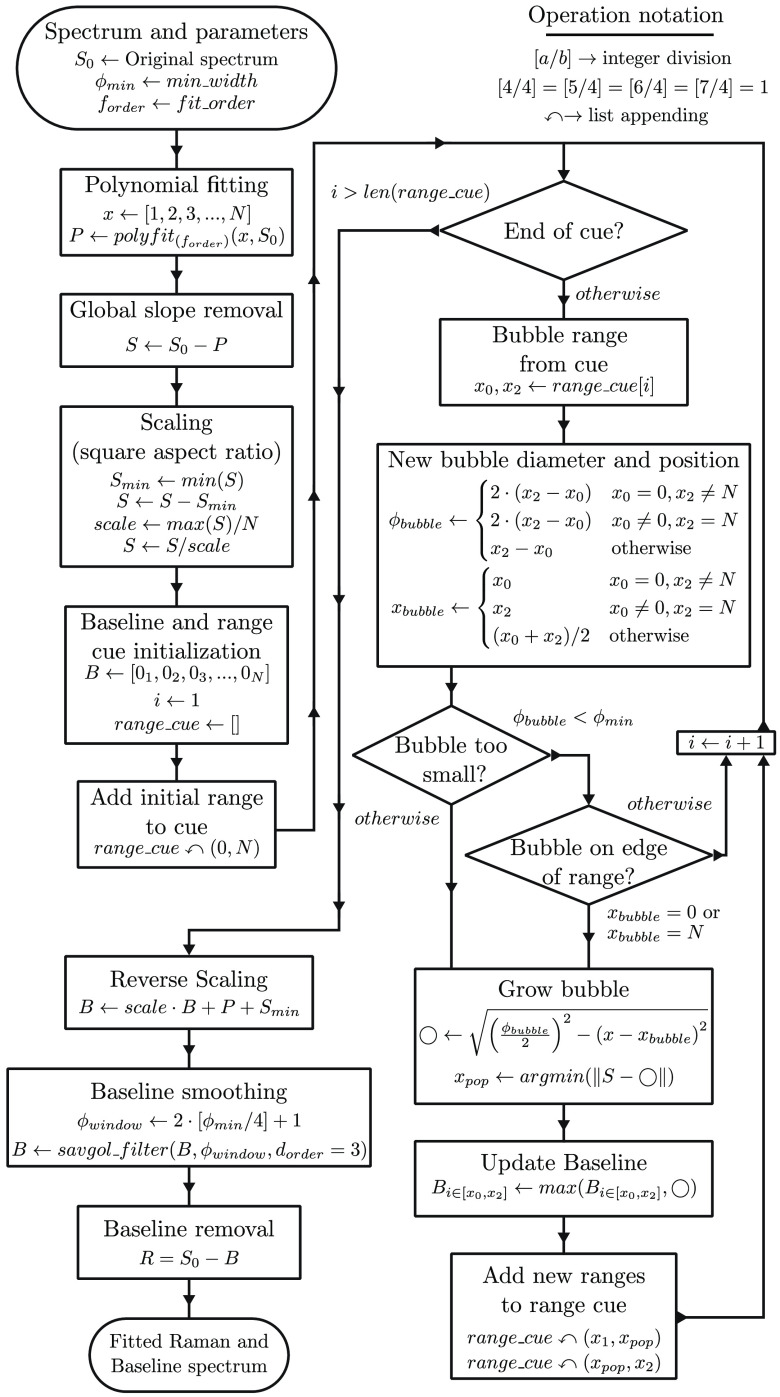
Flowchart of the BubbleFill baseline removal algorithm. The algorithm inputs are: (a) A raman signal ($S_{0}$) represented as a vector of length N; (b) the minimum width of the bubbles allowed to grow ($\phi_{\min}$); and (c) the order of the polynomial fit used to remove the global slope of the signal ($f_{\text{order}}$). First, the global slope of the signal ($S_{0}$) is removed using a polynomial fit and the signal intensity is normalized to obtain a square aspect ratio ($\min(S_{0})=0$, $\max(S_{0})=1$). Then, bubbles of increasingly smaller size are grown underneath the signal and the baseline fit (B) is iteratively updated. Once the bubble growth loop is completed, the square aspect ratio normalization is reversed and the baseline fit is smoothed using a Savitzky–Golay filter. The output Raman signal is computed as the subtraction of the baseline fit to the input signal.

**Fig. 5:**
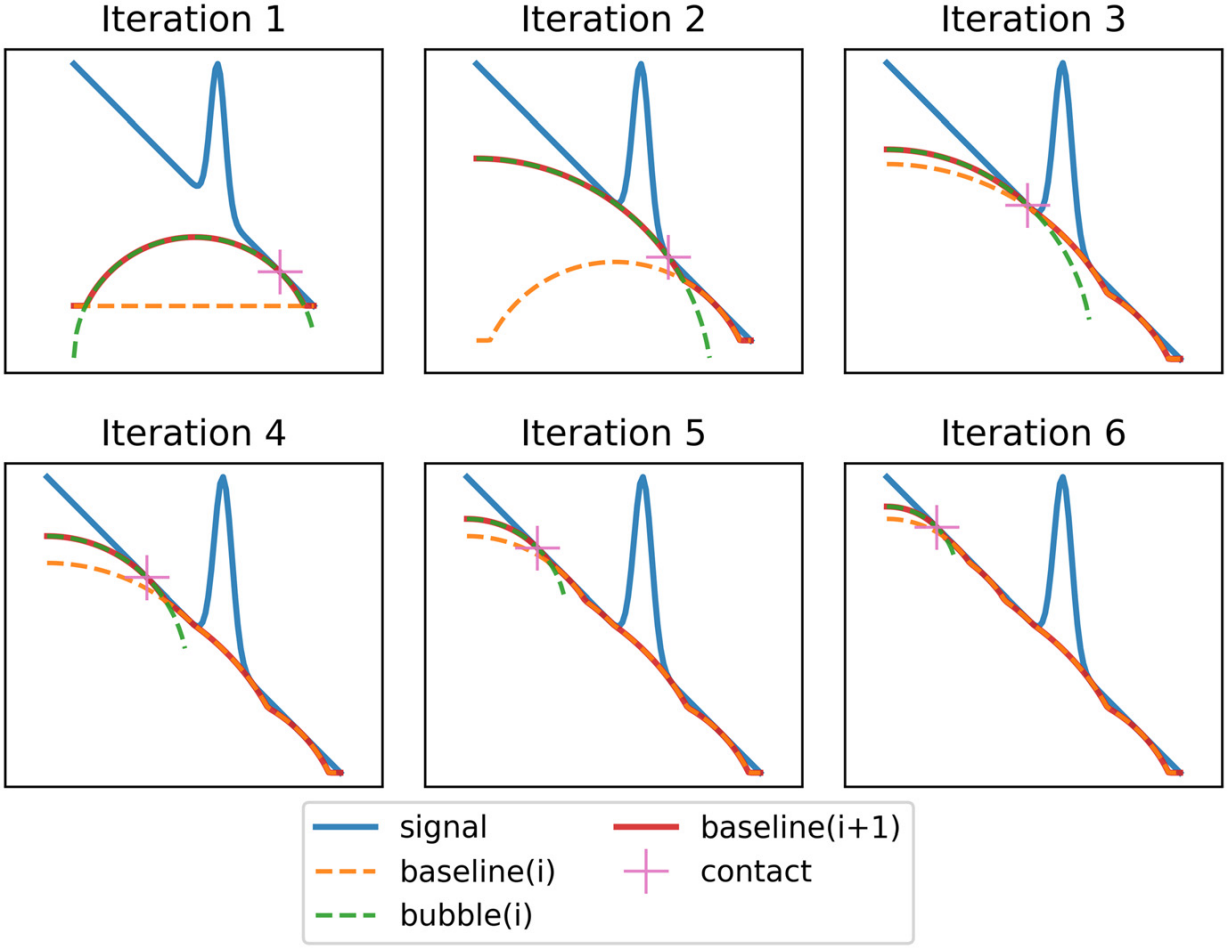
First six iterations of the bubble growth and baseline update iterative process that is at the core of the BubbleFill algorithm. Only the first six iterations are displayed, resulting in a small, obtuse angle toward the right side of the peak. Additional iteration reveals that a small bubble is eventually grown in that region and significantly improve the baseline fit. The Savitzky–Golay filter applied to the baseline fit after the bubble growth process further smooths possible remaining sharp angles.

Inspired by the approach of Perez-Pueyo (MorphBR), we developed a new algorithm based on morphological processing that results in a smoother baseline fit, introduces fewer artifacts and for which fitting parameters can be tuned to different levels across the x-axis. We named this new algorithm BubbleFill because the fitting process uses circular bubbles to fill the region underneath the spectrum. A detailed flowchart of the algorithm is shown in Fig. 4 and an illustration of the bubble growth loop is presented in Fig. 5. First, the overall slope of the spectrum is removed using a linear fit and the result is scaled for the x-axis and y-axis to span the same range (square aspect ratio). The baseline estimate is initialized at 0 over the entire x-axis. Then, circular bubbles are iteratively grown under the scaled spectrum, starting with a bubble of diameter equal to the spectrum’s length aligned on the center of the x-axis. The bubble pops when it reaches the spectrum, and two new bubbles start to grow on each side of the contacting point. A bubble’s diameter and alignment depend on the x-axis region where it is grown. After every bubble has popped, the new baseline estimate is updated as the maximum value between the bubble and the current baseline. This process repeats until every new bubble has reached the critical minimum diameter specified as a tuning parameter. Finally, the baseline estimate is smoothed using a Savitzky–Golay filter and scaled back to the original spectrum y-axis range. The final Raman signal is then obtained by the subtraction of the baseline estimate to the original spectrum. The only tuning parameter corresponds to the smallest allowed bubble diameter. The smaller they are allowed to grow, the more aggressive is the baseline fitting process and vice versa. Additionally, it is possible to specify different bubble diameters across the x-axis, effectively achieving a different degree of sensitivity over different regions of the input signal.

### 2.6 X-axis Calibration

For the same reason that the y-axis calibration of a Raman instrument should ideally not be made using a calibration light source, the x-axis Raman shift needs to be computed from a reference sample’s spectrum. Reference samples used for this purpose should have a Raman to baseline ratios (RBR) that is the ratio between the intensity of the tallest Raman peak to the maximum of the baseline signal, of at least 0.2. Additionally, it is preferable to use samples featuring narrow Raman peaks that can be easily identified with an automatic peak-finding tool, such as SciPy’s find_peaks() function. Acetaminophen and Nylon are two reference samples well suited for this purpose. They both feature narrow peaks that are uniformly spread over the 0 to $2000\;{\mathrm{cm}}^{-1}$ region, have an RBR ratio above 0.5, are shelf stable while not requiring tedious maintenance and can easily be brought into sterile environments.

To calibrate a Raman spectrometer’s x-axis, a spectrum is measured from a chosen reference sample, then the spectrum is processed using the steps described in Secs. 2.1–2.5. The camera pixel location index of the most prominent Raman peaks are identified using a peak-finding tool [e.g., SciPy’s find_peaks() function]. Finally, a polynomial fit of order 2 or 3 is used to create a map conversion between camera pixel index and Raman shifts. An important note, when calibrating the x-axis for machine learning applications, is that it is critical to interpolate every spectrum over a common x-axis. Otherwise, there is no guarantee that all spectra are expressed in the same vector space or, in other words, that the intensity vectors across spectra correspond to the same wavelength/energy.

### 2.7 Smoothing and Normalization

Smoothing and normalization are highly dependent on the end use of a Raman spectrum. When spectra are intended for visual analysis, they are smoothed using an average moving filter and normalized so that the resulting spectrum’s minimum is 0 and maximum, or intensity at a chosen Raman shift, is 1. This type of normalization helps with visual comparison and assessment of spectra acquired on samples that cover a wide range of absorption and scattering. When spectra are used in machine learning applications, however, they are not smoothed and are normalized using the standard normal variate (SNV) method. The SNV normalized ($s^{⋆}$) transformation of a signal (s) is given as

$$s_{i\in [0,N]}^{⋆}≔\frac{s_{i}-\overset{¯}{s}}{\frac{1}{N}\underset{i}{\sum }{\left(s_{i}-\overset{¯}{s}\right)}^{2}},$$ (2)

where the normalized signal has a mean of 0 and a standard deviation of 1.

### 2.8 Spectrum Quality Factor

The definition of a general spectra quality factor is necessary for comparison and ranking of signals from different datasets. However, common metrics such as the SNR or the RBR tend to be highly dependent on the instrument (spectrometer slit and resolution and detector efficiency) and acquisition parameters (excitation power, and exposure time). Because of this, Raman signals measured from different sample types or using different systems tend to have vastly different SNR and RBR. Often, a top-quality spectrum from one dataset would have an SNR and RBR smaller than every spectrum from another dataset. It is therefore impracticable to use either metric for general spectrum quality comparison across multiple datasets or instruments. Instead, the quality of the Raman spectra presented in this work was measured using the average signed squared intensity (ASSI) as defined as

$$\mathrm{ASSI}≔\frac{1}{N}\overset{N}{\underset{i=1}{\sum }}\;sgn(r_{i}^{⋆})\cdot r_{i}^{⋆2},$$ (3)

where $r^{⋆}$ is an SNV normalized Raman spectrum and sgn(x) is the sign function of x, that is −1 or 1 whether x is negative or positive. Given this definition, the ASSI of an arbitrary Raman signal is bound between −1 and 1. Because an SNV-transformed signal’s average is 0, squaring the Raman intensity is necessary for the ASSI computation sum to return a non-zero value. Additionally, this non-linear scaling and the use of the sign function favors large intensity peaks and penalizes signals that have intensity drops below the signal average. In summary, if a signal contains few large and narrow peaks, its ASSI will be large, whereas if a signal contains many small and broad peaks, its ASSI will be small. Finally, a signal that contains only stochastic noise will have an ASSI of 0.

## 3 Results and Discussion

Two different complementary approaches were used for the validation of ORPL. First, a module for the numerical generation of synthetic spectra was implemented as part of the library. This module enables the creation of synthetic benchmark spectra that can be used for quantitative testing of the novel BubbleFill baseline removal algorithm. Then, experimental datasets from previous studies have been compiled and uniformized in a single dataset. The uniformization consisted in the conversion of all data files into the .json open standard file format and the bundling of acquisition metadata as object properties for each spectrum. The compound dataset was used for validation of the capabilities of ORPL for the processing of spectra from different biological sample types (including different tissue types or similar tissues with different sample preparation) across different systems. Here, validation served to confirm that the BubbleFill algorithm addresses current limitations of other baseline removal algorithms and that the processing workflow implemented in ORPL enables the recovery of Raman spectra featuring vibrational bands commonly expected in biological samples.

### 3.1 Comparison of Baseline Removal Algorithms: Synthetic Tool

Synthetic spectra ($S_{i}$) were modeled as a combination of three signal components: Raman ($R_{i}$), baseline ($B_{i}$) and noise ($N_{i}$). Generation of benchmark signals in this manner allows a fine control on the desired SBR and SNR. Additionally, outputs given by a baseline removal algorithm can be compared to the original signal components with which the input spectrum was generated to compute overall fitting error. However, the Raman and baseline components used for the generation of synthetic spectrum need to be independent of the baseline removal algorithm to be tested to limit possible biases. The formula used to generate synthetic spectra is

$$S_{i}=\frac{sbr\cdot R_{i}+B_{i}}{\max(sbr\cdot R_{i}+B_{i})}+N_{i},$$ (4)

where the index i runs from 1 to N, that later being the total number of bins composing each spectrum.

**Fig. 6:**
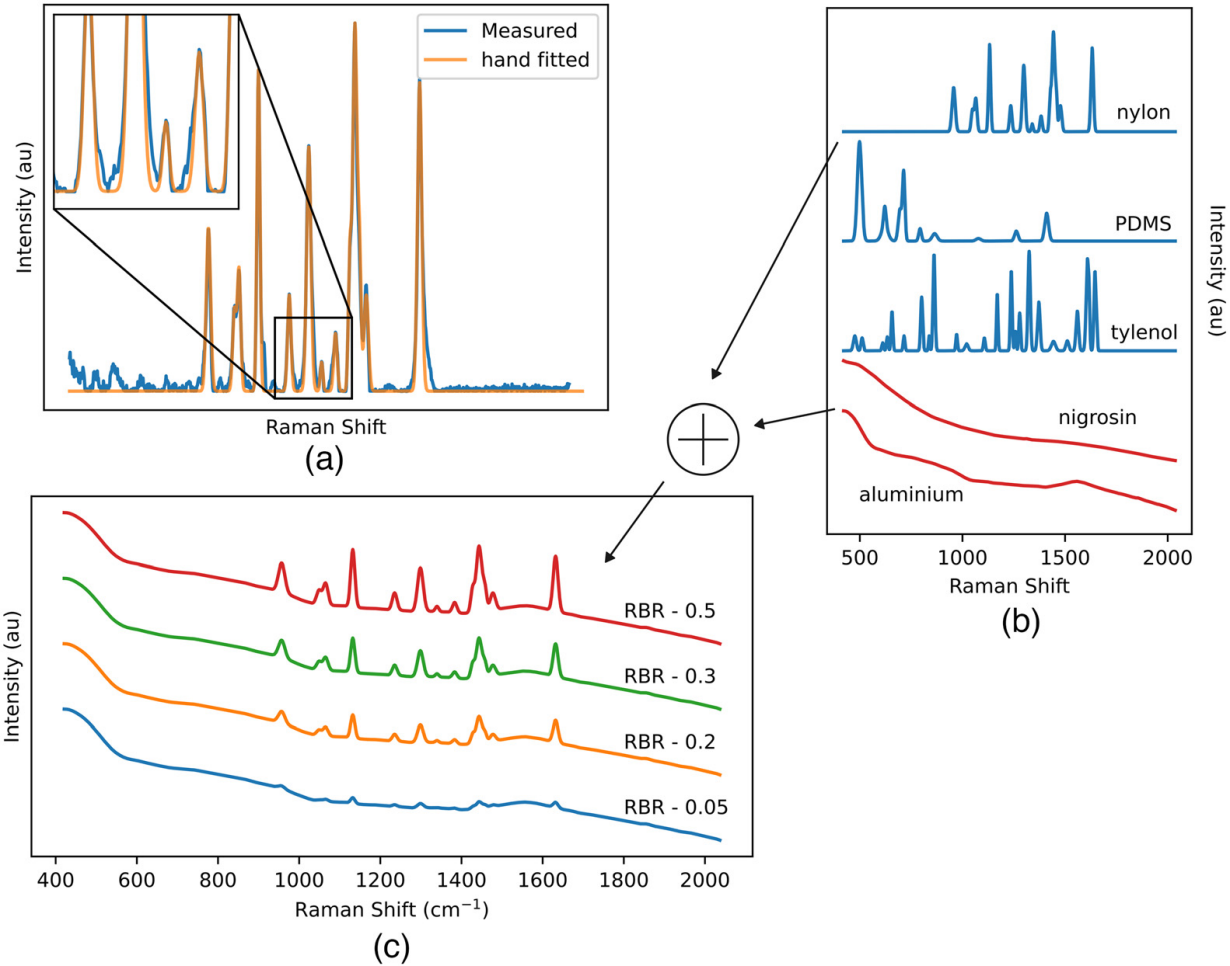
Baseline algorithm benchmark with synthetic spectrum generated from the aluminium baseline and acetaminophen Raman with a RBR of 0.05. (a) Manual fitting of a signal composed of Gaussian peaks over a experimental spectrum. (b) Synthetic benchmark spectra can be created by combining a Raman signal component (nylon, PDMS or tylenol) and a baseline signal component (nigrosin or aluminium). (c) The Raman to Baseline Ration can be adjusted to be representative of different tissue types or applications.

The Raman component used for generation of benchmark spectra were experimentally measured on acetaminophen, Nylon and PDMS samples using a point probe system^44^ and were processed using the aforementioned workflow. The experimental Raman spectra were hand-fitted as a superposition of Gaussian curves [Fig. 6(a)] to ensure only clean Raman peaks remained, and to limit eventual biases introduced by the baseline removal algorithm. For the baseline component, spectra were measured on aluminium and nigrosin. As neither of the signals featured noticeable Raman peaks, it was concluded that their spectral responses consisted essentially of pure fluorescence (baseline) signals. These experimental baselines were smoothed using a 50-pixel wide average moving filter to further remove residual traces of noise resulting in the Baseline components ($B_{i}$) used for the generation of benchmark spectra. Together, the two baseline components and three Raman components [Fig. 6(b)] enable the creation of a wide variety of possible synthetic spectrum suited for use as quantitative benchmark tests for baseline removal algorithm validation and optimization [Fig. 6(c)]. Finally, the noise component ($N_{i}$) is generated following a normal distribution with an average of 0 and a specified standard deviation.

The metric chosen to evaluate the performance of the different baseline removal algorithms implemented in ORPL is the normalized mean squared error (nMSE). The nMSE between a Raman signal computed from a baseline removal algorithm ($R_{i}^{c}$) and the Raman target ($R_{i}^{t}$) used in the generation of a benchmark signal is defined as

$$n\mathrm{MSE}≔\frac{\underset{i}{\sum }{\left(R_{i}^{t}-R_{i}^{c}\right)}^{2}}{{\left(\underset{i}{\sum }R_{i}^{t}\right)}^{2}}.$$ (5)

### 3.2 Baseline Removal Benchmark without Noise

A benchmark spectrum is generated using the acetaminophen Raman and aluminium baseline components with an SBR of 0.05. The spectrum’s baseline is removed with the BubbleFill, MorphBR and iModPoly algorithms and their respective Raman outputs are compared to the target. The results presented are the optimal fit of each algorithm (smallest nMSE) obtained by sweeping the possible range of value for their respective tuning parameter. This method is used to compare baseline removal algorithms in a ‘best-case scenario’ leveled playing field.

**Fig. 7:**
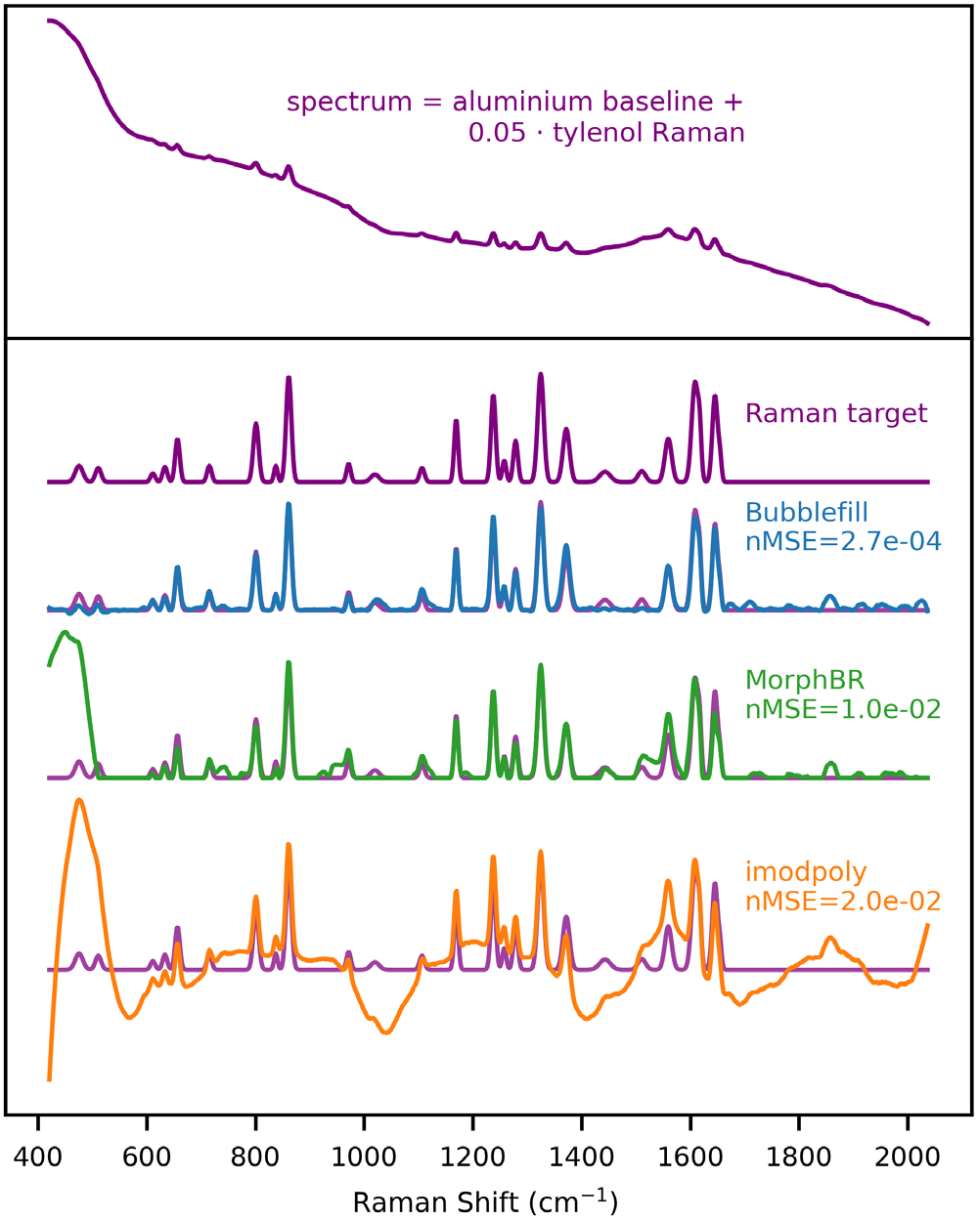
Baseline algorithm benchmark with synthetic spectrum generated from the aluminium baseline and acetaminophen Raman. Signal-to-noise : 0.05. Noise standard deviation : 0.

**Fig. 8:**
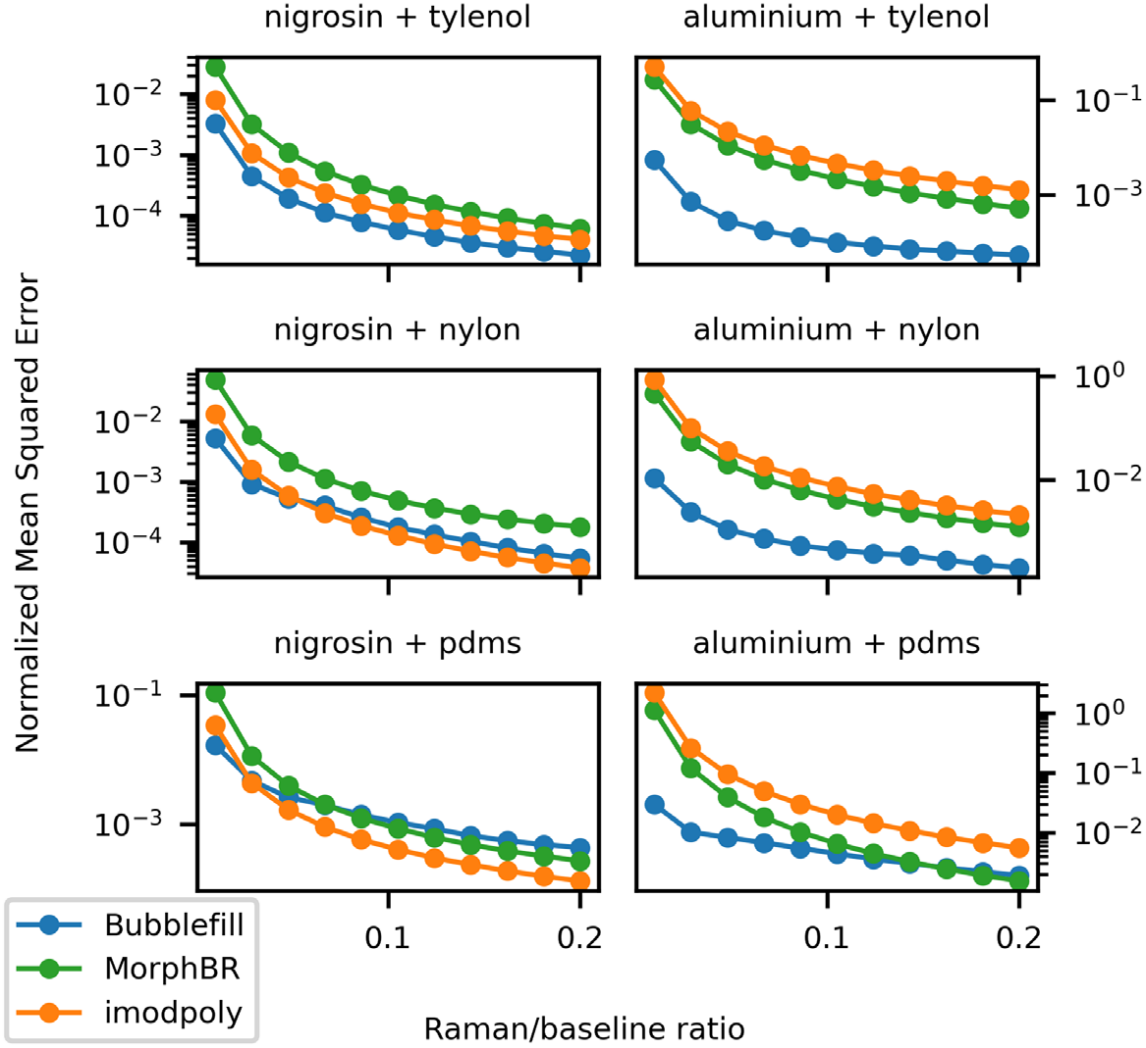
nMSE as a function of the RBR for the three algorithms tested. Each graph is for spectra generated with a different combination of baseline and Raman signal components.

This first example highlights the shortcomings of polynomial fitting methods when removing baselines that feature “localized bumps,” such as the ones seen in many biological samples (Fig. 7). Even though not all biological sample feature a baseline as difficult to remove as aluminium, localized bumps can still be introduced in the measured signal when acquisitions are made over an aluminium substrate. This is relevant and problematic because aluminium slides are becoming an increasingly popular and cheaper alternative to calcium fluoride (${\mathrm{CaF}}_{2}$) slides for Raman microscopy.^11^^,^^41^^,^^45^ The Raman spectrum given by the BubbleFill algorithm closely matches the target except for the two smaller peaks near $1500\;{\mathrm{cm}}^{-1}$. In most instances, using a different tuning (e.g., larger bubbles) would result in fewer small peaks being removed at the cost of a worse baseline fit. This is the general trade-off when tuning any baseline removal algorithm. However, with BubbleFill, it is possible to use a different tuning for different regions of the spectrum. For instance, the bubbles grown in the 1400 to $1600\;{\mathrm{cm}}^{-1}$ region could be much larger than for the rest of the spectrum. This would result in a similar baseline fit, but the small peaks near $1500\;{\mathrm{cm}}^{-1}$ would be preserved. This feature is, to our knowledge, unique to BubbleFill and could lead to significant improvements to baseline removal in some applications. Furthermore, additional testing with different combinations of baseline and Raman components and for different SBRs indicate that BubbleFill outperforms other tested algorithms in numerous instances (Fig. 8). The largest difference in performance was observed for spectra generated with the aluminium baseline and acetaminophen or nylon Raman components. In these cases, BubbleFill has an nMSE nearly two orders of magnitude smaller than the second-best performing algorithm. It fell behind only for spectra generated with nigrosin baseline and nylon or PDMS Raman components where it still remained the best option when the RBR approached 0.

The different algorithms tested in this work along with the results presented in this section are summarized in Table 1.

**Table 1:** Baseline algorithm comparison summary. Execution time was measured on a benchmark spectrum generated with the Nylon Raman, aluminium baseline, Raman/baseline ratio of 0.5 and noise standard deviation of 0.1.

Algorithm tested	General performance	Tuning parameters	Intuitiveness of tuning parameters	Noticeable shortcomings	Execution time on a signal of size 1000
iModPoly	−	Order of polynomial fit, precision target	−	Fails to fit non-polynomial baselines.	2.6 ms±7.65 μs
MorphBR	+	Morphological filter window size	−	‘Jagged staircase’ effect in the fitted baseline.	727 μs±1.71 μs
				Introduces a hill in the output Raman near the origin of the x-axis.	
BubbleFill	++	Minimal bubble width	+	—	743 μs±2.44 μs

### 3.3 Baseline Removal Benchmark with Noise

Baseline removal algorithms have been tested on spectra with noise to confirm that the results shown thus far can translate to real-world applications. However, the method of comparing algorithms’ performance via the mean squared error becomes impractical when noise is added to the input signal. The problem is that the MSE between an algorithm’s computed Raman and the target is overwhelmingly correlated with the added noise itself. This makes the computed MSE seemingly identical between the three algorithms in every scenario tested, even when differences remained visually noticeable in the respective outputs. Instead of comparing nMSE across the different algorithms on noisy spectra, two examples have been chosen to illustrate tendencies also observed in the experimental datasets presented in the following section. In both examples, the input signal was generated using baseline and Raman components as described before, but with the addition of noise. The noise signal added follows a normal distribution with average 0 and standard deviation of 0.01, i.e., 1% of the baseline’s maximum.

**Fig. 9:**
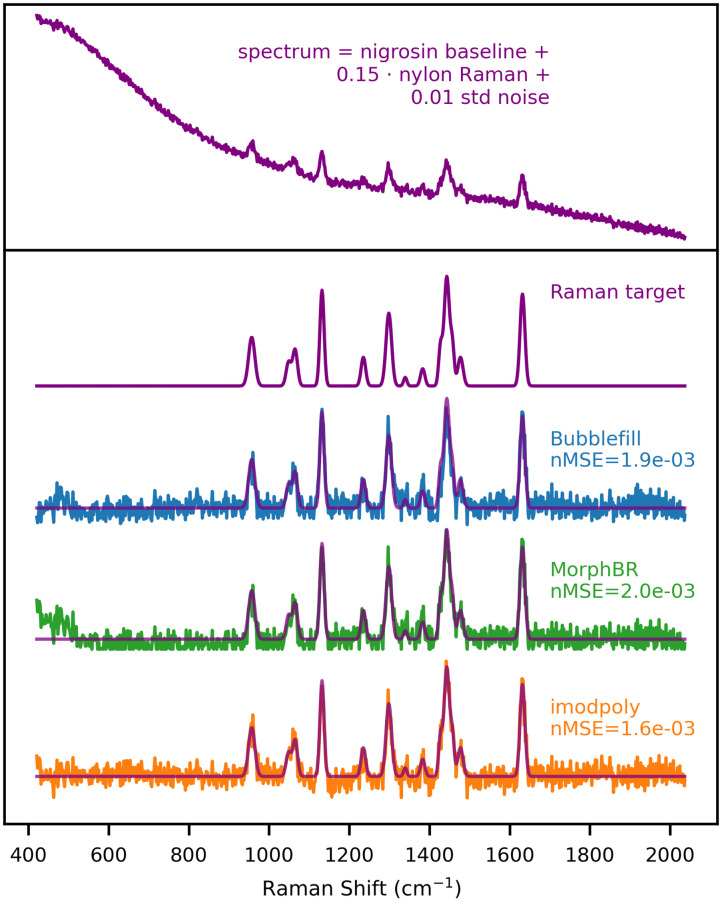
Baseline algorithm benchmark with synthetic spectrum generated from the nigrosin baseline and nylon Raman. RBR : 0.15. Noise standard deviation : 0.01.

Figure 9 shows the baseline removal benchmark on a spectrum generated from nigrosin and nylon with Raman-to-noise ratio of 0.15. This example was chosen because it is a best-case scenario for the iModPoly algorithm—the nigrosin baseline is smooth, and the nylon peaks are easily identifiable and located toward the center of the spectrum. Yet, both BubbleFill and MorphBR performed similarly to iModPoly.

**Fig. 10:**
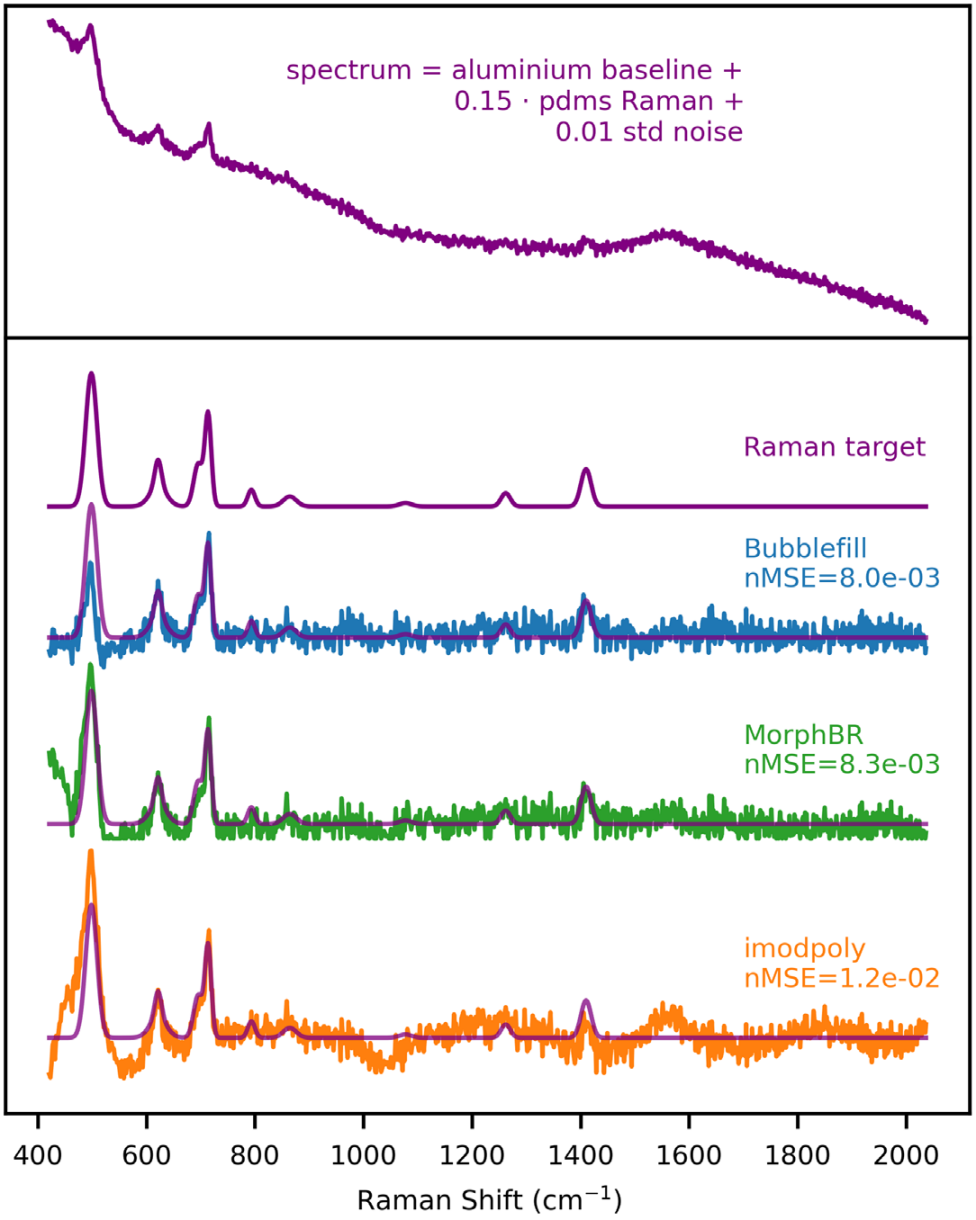
Baseline algorithm benchmark with synthetic spectrum generated from the aluminum baseline and PDMS Raman. RBR : 0.15. Noise standard deviation : 0.01.

Figure 10 shows the baseline removal benchmark on a spectrum generated from aluminium and PDMS. Similarly to the tests performed on signals without noise, iModPoly failed to correctly remove the aluminium baseline while BubbleFill and MorpthBR managed a near-perfect recovery of the Raman target except for a small discrepancy (shared by every algorithm) for the first peak near $500\;{\mathrm{cm}}^{-1}$. In general, none of the algorithm’s performance was significantly affected by the addition of noise to the input spectrum.

### 3.4 Testing ORPL with Different Experimental Datasets

**Fig. 11:**
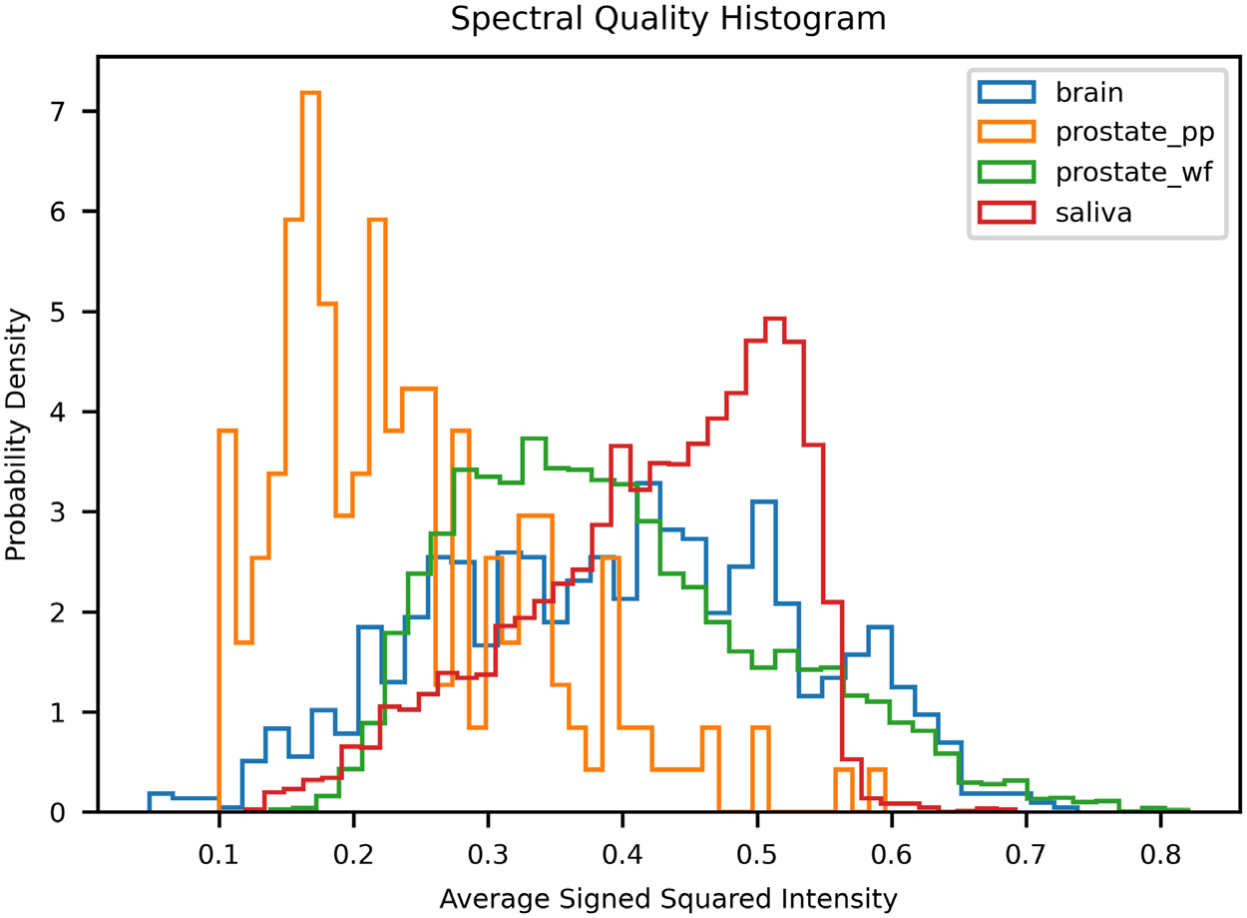
Spectral quality histogram of the experimental datasets (brain, prostate Point Probe, prostate Wide-Field and saliva) measured with the ASSI quality metric.

We tested the capability of ORPL on four datasets acquired with three different Raman platforms to cover a wide range of sample types, instruments, and sample preparation methods. The first dataset (1719 spectra) consists of *in vivo* brain tissue spectra measured with the hand-held probe from Reveal Surgical and provides a good reference for spectra acquired in a surgical workflow. The second dataset (524 spectra) consists of in vivo and ex vivo prostate tissue spectra measured with a custom lab-built system using a commercial EmVision LLC handheld probe. The third (8670 spectra) and fourth datasets (7774 spectra) consist of paraffin-fixed prostate tissue slices and dried saliva samples respectively and were measured with a commercial Renishaw Raman microscope as 3D Raman maps. Combined, the four datasets amount to a total of 18,687 individual acquisitions (not counting repeated accumulations) that cover a wide range of signal-to-background and SNR as confirmed by the spectral quality of each signal measured using the ASSI metric defined earlier (Fig. 11).

**Fig. 12:**
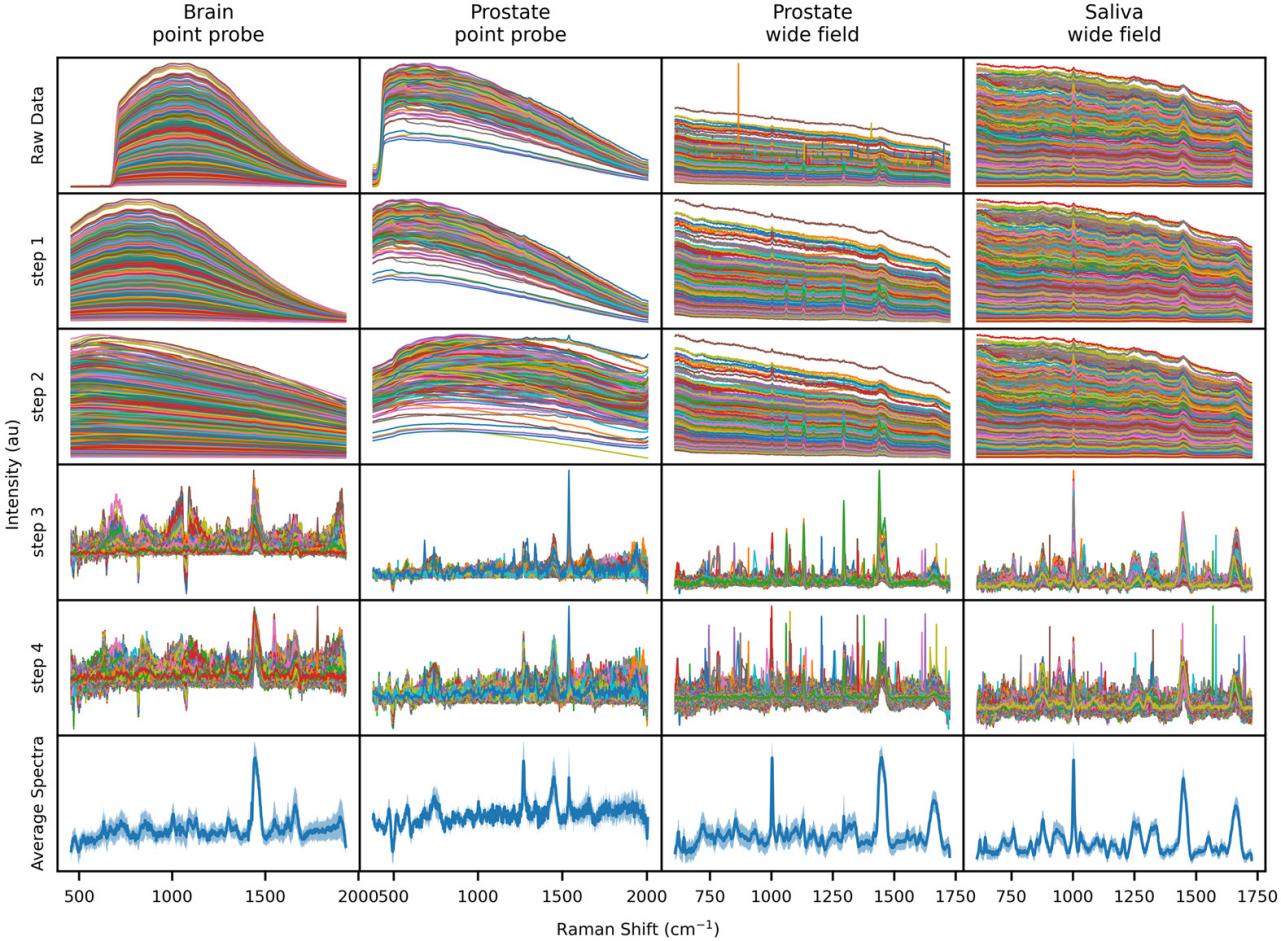
Raw data: measured raw spectra from instruments with accumulations combined. Step 1: Truncation (for datasets 1 and 2) and cosmic ray removal. Step 2: Background removal (for datasets 1 and 2) and calibration of x and y axis. Step 3: Baseline removal with BubbleFill. Step 4: SNV normalization. Average Spectra: average spectra computed from step 4 results with +- standard deviation zone represented as shadow.

The raw data (first row of Fig. 12) shows that the spectra of each dataset covered nearly the entire dynamic range of the acquisition instruments. This is frequent with biological samples and is explained by the large variability of fluorescence signal strength, absorption, and scattering. Nevertheless, Raman pre-processing using the workflow presented in this work and the BubbleFill algorithm for the removal of intrinsic fluorescence (step 3 of Fig. 12) resulted in averaged spectra with small deviations. Many common Raman active bands can be identified and are common across all datasets, including vibrational modes typically found in proteins and lipids.

**Fig. 13:**
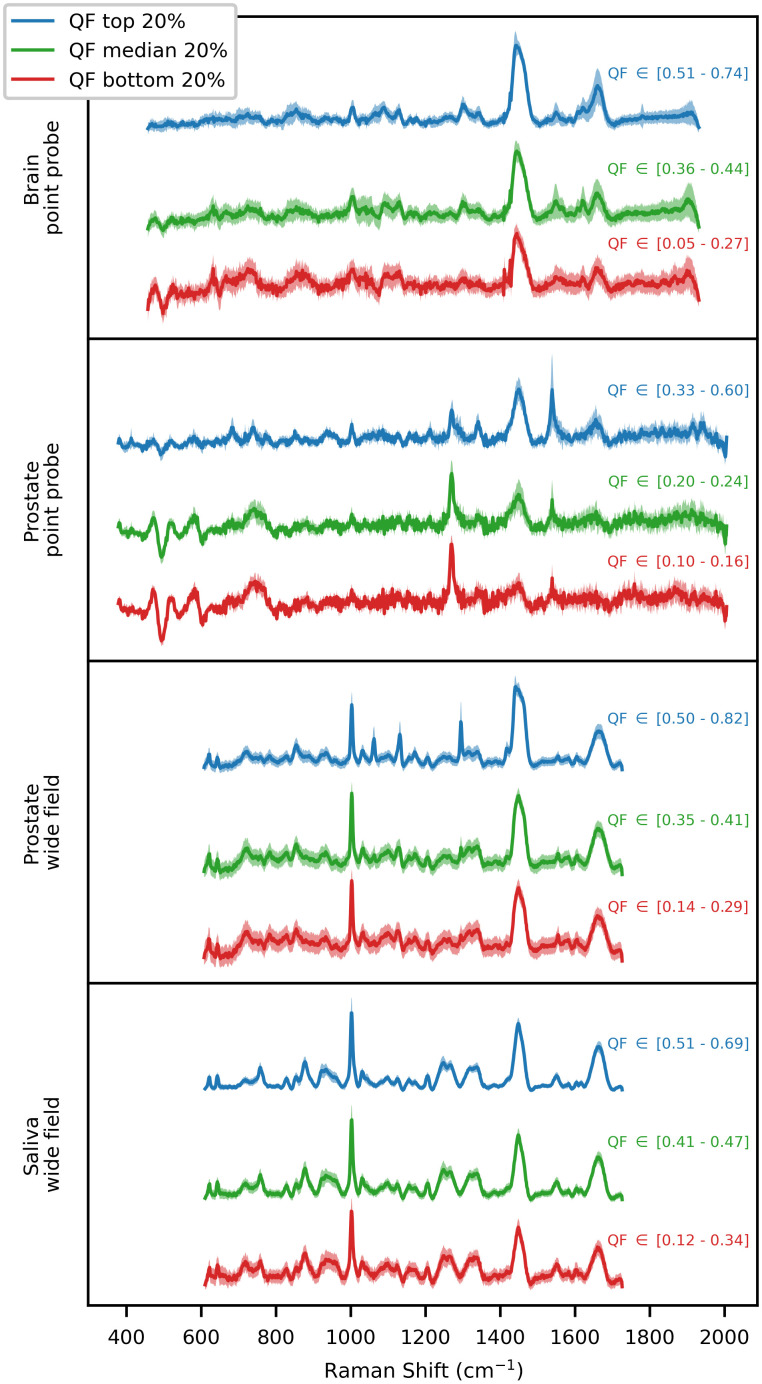
Average pre-processed spectra of each experimental tested datasets clustered in high (top 20% ASSI), average (middle 20% ASSI) and low (bottom 20% ASSI) quality.

Finally, spectra of all datasets were clustered in groups of high, average, and low quality based on the ASSI metric (Fig. 13). As the quality increased, the standard deviation became smaller, converging toward the average spectrum. This behavior was observed for all datasets. Furthermore, some specific Raman bands (peak at $1300\;{\mathrm{cm}}^{-1}$ for the prostate wide field dataset) gained in intensity while artifacts (400 to $600\;{\mathrm{cm}}^{-1}$ for the prostate point probe dataset) disappeared. These results indicate that the ASSI metric has the potential to be used in machine learning applications to discard low-quality spectra based on a threshold or as a general signal quality metric during acquisition to facilitate troubleshooting of instrument and software.

## 4 Conclusion

In conclusion, we developed and released a python package under the MIT license that implements the necessary tools for Raman spectra pre-processing. Most notably, the ORPL package includes the novel BubbleFill algorithm intended for the removal of autofluorescence baselines. We validated BubbleFill using a combination of numerical benchmarks based on synthetic spectra and real-world experimental data from previous studies. Comparative benchmark results revealed that BubbleFill performed better especially for the removal of an aluminium baselines, which is of critical importance in many of our group’s studies, or as well as other commonly used algorithms. Although these results might not generalize to every possible baseline shapes, the quantitative comparison methodology presented in this work can be extended to include a larger variety of fluorescence and Raman responses. This makes it ideal for selecting and tuning baseline removal algorithms for specific applications while limiting user biases. Finally, we combined the Raman acquisitions of previous studies in a single dataset which is, to our knowledge, the largest and most varied Raman dataset compiled for the purpose of testing and validating pre-processing. This data was used to validate the ORPL package on signals covering a wide range of signal-to-noise and SBRs representative of the biological application landscape. In the future, additional modules will be added to ORPL to address other critical challenges such as spectral unmixing and peak analysis tools and chemometric analysis. It is our hope that this package be used as a stepping stone enabling a more open and uniformed pre-processing methodology across the Raman research and clinical spectroscopy scientific communities.

## Code, Data, and Materials Availability

The ORPL package is hosted on the python package index (PyPi) as an open-sourced project distributed under the MIT license and the name ORPLIB.^46^ The source code is available on github at https://github.com/mr-sheg/orpl. The data and materials information that support the findings of this study are available from the corresponding author upon reasonable request.
